# Integrated microRNA and transcriptome profiling reveals a miRNA-mediated regulatory network of embryo abortion under calcium deficiency in peanut (*Arachis hypogaea* L.)

**DOI:** 10.1186/s12864-019-5770-6

**Published:** 2019-05-21

**Authors:** Hua Chen, Qiang Yang, Kun Chen, Shanshan Zhao, Chong Zhang, Ronglong Pan, Tiecheng Cai, Ye Deng, Xingjun Wang, Yuting Chen, Wenting Chu, Wenping Xie, Weijian Zhuang

**Affiliations:** 1 State Key Laboratory of Ecological Pest Control for Fujian and Taiwan Crops, Fujian Agriculture and Forestry University, Fuzhou, 350002 Fujian People’s Republic of China; 2 Fujian Provincial Key Laboratory of Crop Molecular and Cell Biology, Fujian Agriculture and Forestry University, Fuzhou, 350002 Fujian People’s Republic of China; 3 College of Plant Protection, Fujian Agriculture and Forestry University, Fuzhou, 350002 Fujian People’s Republic of China; 4 Department of Life Science and Institute of Bioinformatics and Structural Biology, College of Life Science, National Tsing Hua University, Hsin Chu, 30013 Taiwan; 5 Biotechnology Research Center, Shandong Academy of Agricultural Sciences, Shandong Provincial Key Laboratory of Crop Genetic Improvement, Ecology and Physiology, Jinan, 250100 People’s Republic of China

**Keywords:** Peanut, miRNA, Embryo abortion, Calcium, Hormone

## Abstract

**Background:**

Peanut embryo development is a complex process involving a series of gene regulatory pathways and is easily affected by various elements in the soil. Calcium deficiency in the soil induces early embryo abortion in peanut, which provides an opportunity to determine the mechanism underlying this important event. MicroRNA (miRNA)-guided target gene regulation is vital to a wide variety of biological processes. However, whether miRNAs participate in peanut embryo abortion under calcium deficiency has yet to be explored.

**Results:**

In this study, with the assistance of a recently established platform for genome sequences of wild peanut species, we analyzed small RNAs (sRNAs) in early peanut embryos. A total of 29 known and 132 potential novel miRNAs were discovered in 12 peanut-specific miRNA families. Among the identified miRNAs, 87 were differentially expressed during early embryo development under calcium deficiency and sufficiency conditions, and 117 target genes of the differentially expressed miRNAs were identified. Integrated analysis of miRNAs and transcriptome expression revealed 52 differentially expressed target genes of 20 miRNAs. The expression profiles for some differentially expressed targets by gene chip analysis were consistent with the transcriptome sequencing results. Together, our results demonstrate that seed/embryo development-related genes such as *TCP3*, *AP2*, *EMB2750*, and *GRF*s; cell division and proliferation-related genes such as *HsfB4* and *DIVARICATA*; plant hormone signaling pathway-related genes such as *CYP707A1* and *CYP707A3*, with which abscisic acid (ABA) is involved; and *BR1*, with which brassinosteroids (BRs) are involved, were actively modulated by miRNAs during early embryo development.

**Conclusions:**

Both a number of miRNAs and corresponding target genes likely playing key roles in the regulation of peanut embryo abortion under calcium deficiency were identified. These findings provide for the first time new insights into miRNA-mediated regulatory pathways involved in peanut embryo abortion under calcium deficiency.

**Electronic supplementary material:**

The online version of this article (10.1186/s12864-019-5770-6) contains supplementary material, which is available to authorized users.

## Background

Among leguminous species, peanut (*Arachis hypogaea* L.) is the most prominent cash crop because of its protein nutrition and oil production and is widely cultivated in tropical and subtropical regions. Peanut embryo development has a direct impact on yield and quality. Embryo development in peanut, a typical geocarpic plant, is a complex process involving the activity of a series of gene regulatory pathways at both the transcriptional and posttranscriptional levels and is easily affected by a variety of elements in the soil, especially calcium (Ca^2+^). Previous studies have demonstrated that calcium in the soil of the pegging zone is vital for embryo development. Calcium deficiency leads to severely diminishing peanut yield and quality [1, 2]. In addition, calcium deficiency adversely reduces seed viability and germination in subsequent seasons. Severe calcium deficiency in the soil induces early peanut embryo abortion. Different approaches, including differential display reverse transcription PCR (DDRT-PCR) [3], SSH-associated library lift (SSHaLL) [4] and proteomic techniques [5], have been used to elucidate the mechanisms governing calcium regulation in peanut embryo development. However, the molecular basis of peanut embryo development, especially embryo abortion, under calcium deficiency conditions is still unknown.

Studies have shown that small RNAs (sRNAs) play important roles in posttranscriptional gene regulation via target messenger RNA (mRNA) degradation or translation inhibition [6]. Thus far, 35,828 microRNAs (miRNAs) from 223 species have been deposited in miRBase (http://www.mirbase.org/). Recent studies have provided an explosive amount of information on miRNA regulation involvement in various biological processes, including organ development [7–9], phase transitions [10–13], and stress responses [14–17]. Increasing evidence indicate that, in plants, miRNAs regulate seed formation and development. Overexpression of miR397b in *Arabidopsis* increased silique numbers and silique length, resulting in increased seed numbers [18]. Wheat grain filling is correlated with miRNA-mediated gene regulatory networks, and 104 grain filling-associated miRNAs might target a set of genes involved in various biological processes, including the metabolism of carbohydrates and proteins, transcription, cellular transport, cell organization and biogenesis, stress responses, signal transduction, and phytohormone signaling [19]. miRNAs contribute to the control of grain development in barley, notably by the regulation of phytohormone response pathways for abscisic acid (ABA), gibberellins (GAs), auxin and ethylene [20]. In addition, miRNAs can affect seed germination [21, 22] and oil accumulation [23, 24]. Recently, miRNA have also been analyzed in peanut. A number of conserved and novel miRNAs were first identified in the roots, leaves and stems of peanut via high-throughput sequencing technology [25]. Regulatory roles of miRNAs in peanut disease resistance and embryogenesis have thus been proposed [26]. However, there are no reports on miRNA regulation in peanut embryo abortion under calcium deficiency.

To better understand the function of miRNAs in peanut embryo development, this study characterized the expression profiles of miRNAs in peanut embryos at three developmental stages under calcium deficiency and sufficiency. Furthermore, global prediction of miRNA targets in peanut was performed, and target genes were identified. Many of the predicted target genes were involved in plant hormone biosynthesis, signal transduction, plant defense responses, cell proliferation, ubiquitin-mediated proteolysis and floral organ development. These results suggest that miRNAs play an important role in regulating early peanut embryo development. Our findings contribute to uncovering the complex regulatory network that occurs during peanut embryo development, especially embryo abortion under calcium deficiency.

## Results

### Calcium deficiency significantly affects peanut pod development

**Fig. 1:**
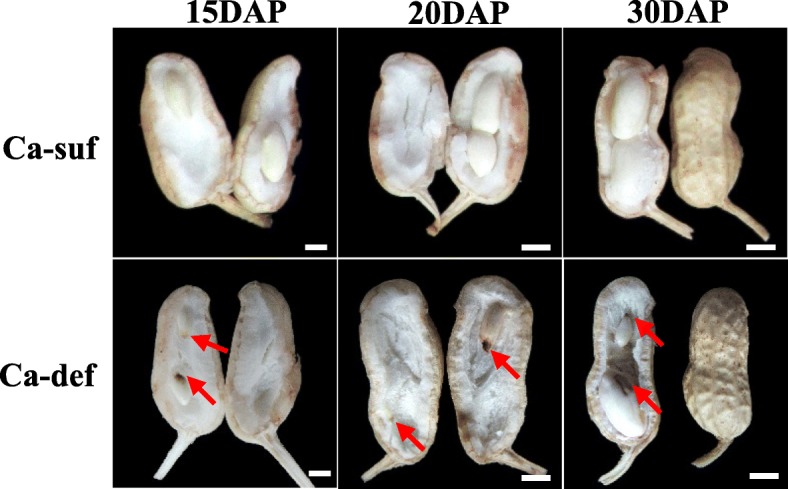
Morphological characteristics of developing peanut embryos under calcium sufficiency and deficiency. Red arrows show seeds starting to rot. The bars indicate 5 mm. Ca-suf, Ca sufficiency; Ca-def, Ca deficiency

Although peanut pods at 15, 20 and 30 days after pegging (DAP) between calcium deficiency and sufficiency conditions did not differ in size (Fig. 1), the seed coat color started to become black, and the embryos tended rot under calcium deficiency, eventually producing empty pods. Calcium sufficiency-treated plants produced fully filled pods (Fig. 1). The biological characteristics examined at harvest indicated that no obvious differences in vegetative growth-related characteristics were observed between calcium deficiency and sufficiency conditions (Table 1). However, the amount and percentage of full pods and the number of rotted pods were significantly different, which manifested as a severe decrease (34.6%) in the dry pod yield of the calcium-deficient peanut plants (Table 1). It is clear that embryo abortion resulting from calcium deficiency could strongly reduce the peanut yield and quality. Therefore, discovering functional genes governing peanut embryo abortion under calcium deficiency conditions is important to elucidate the molecular mechanism underlying peanut seed development, yield and quality formation.

**Table 1:** Differences in growth and development peanut plants under calcium deficiency and sufficiency conditions

Treatment	Height of main stem (cm)	Length of branch stem (cm)	Total branches (Num)	Pod number per plant	Full pod number per plant	Rotted pod number per plant	Percentage of full pods (%)	Number of gynophores	Dry pod weight per plant (g)
Calcium deficiency	25.6	29.6	6.8	19.6	2.6A	9.5A	15.1A	34.9	6.6A
Calcium sufficiency	25.9	30.7	6.3	17.9	14.6B	0B	81.2B	31.9	14.9B

Note: A, B indicates significant difference at 1% level

### sRNA library sequencing results

**Fig. 2:**
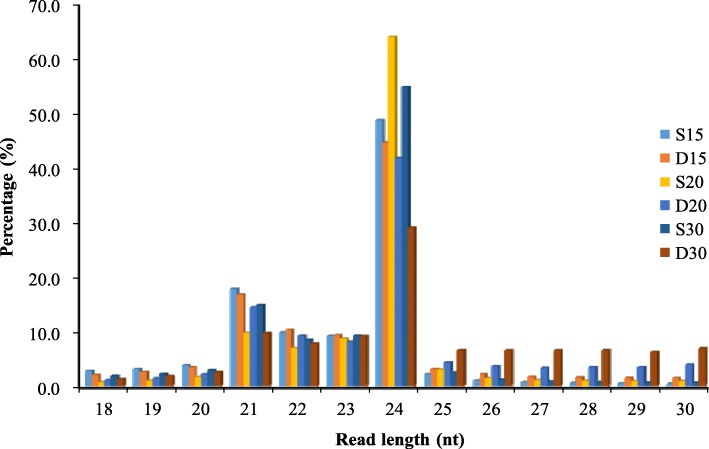
Size distribution of sRNA sequences identified from calcium deficiency- and sufficiency-treated embryo libraries

**Fig. 3:**
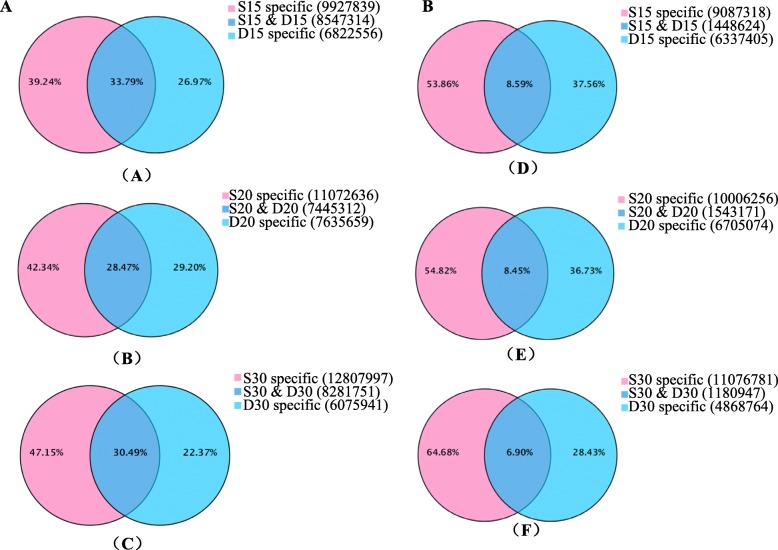
Common and unique sRNAs between calcium deficiency (D15, D20, D30)- and sufficiency (S15, S20, S30)-treated embryo libraries. **a**: total sRNAs, **b**: unique sRNAs. (A) and (D) 15 DAP, (B) and (E) 20 DAP, (C) and (F) 30 DAP

To identify the regulatory roles of miRNAs involved in peanut embryo abortion under calcium deficiency conditions, the sRNAs were analyzed using Illumina sequencing technology in embryos at 15, 20 and 30 DAP under calcium deficiency and sufficiency conditions; more than 20 million reads were generated. After the removal of adaptor sequences, RNAs shorter than 18 nucleotide (nt) and polyA sequences, more than 17 million reads were generated from most of the samples (Additional file 5: Table S1). These total reads contained miRNA, ribosomal RNA (rRNA), small nuclear RNA (snRNA), transfer RNA (tRNA), small nucleolar RNA (snoRNA), and unannotated sequences (Additional file 5: Table S1). However, only 40%~ 50% of the clean reads mapped perfectly to the peanut genome (Additional file 6: Table S2). The clean reads were aligned with the reference genome (https://www.peanutbase.org/) for miRNA identification and here no mismatch between small RNA and the genome sequence was allowed. The reference genomes were created from the wild peanuts *Arachis duranensis* and *Arachis ipaensis*, which were regarded as ancestors of cultivated peanut. That maybe the reason why the mapped reads were low. The correlation coefficients of the samples are shown in Additional file 1: Figure S1. sRNAs that were 21–24 nt in length were dominant in all six libraries, accounting for more than 80% of the total sRNAs in the S15, D15, S20 and S30 libraries; 73.51% in the D20 library; and 55.69% in the D30 library (Fig. 2). Among these sRNAs, those that were 24 nt in length were most abundant (> 40% of the total reads, except in D30), followed by those that were 21 nt in length (~ 14%). These results were consistent with that concerning tomato fruit [27], somatic embryogenesis in citrus [28], and soybean root hairs [29] as well as those of previous studies in peanut [25, 30] but were different from those of *Astragalus chrysochlorus* [31]. Interestingly, the proportion of 24 nt reads (29.05%) in D30 was significantly lower than that in other libraries, while the proportions of 25–30 nt reads progressively increased in D15, D20 and D30 (Additional file 7: Table S3). There were 27.0, 29.2, and 22.4% total sRNAs and 37.6, 36.7 and 28.4% unique sRNAs specifically in calcium deficiency-treated samples at 15, 20 and 30 DAP, respectively. Samples with 39.2, 42.3 and 47.2% total sRNAs and 53.7, 54.8 and 64.7% unique sRNAs were specifically found in the sufficiency conditions; there were ~ 30% total (~ 8% unique) sRNAs common to the samples of both conditions (Fig. 3). After the removal of rRNA, tRNA, snRNA, and snoRNA sequences as well as repeat and exon sequences, the remaining unique reads were used for miRNA predictions.

The first nucleotide of the 5′ end of a miRNA interacts with a specific AGRONAUTE (AGO) protein [32]. To determine whether a bias exists in the 5′ nucleotide of the peanut embryo sRNAs, the distribution of the first nucleotide of each sRNA sequence was calculated relative to the length of the sRNA (Additional file 2: Figure S2 and Additional file 8: Table S4). Uridine, which is a characteristic of sRNA that associates with AGO1, was more abundant than the other three possible nucleotides at the first nucleotide in the sRNAs that were 19–22 nt in length; the percentages were 80, 60, 59 and 72% (Additional file 2: Figure S2 and Additional file 8: Table S4). In contrast, adenosine (45%) was the most abundant 5′ nucleotide of the sRNAs that were 24 nt in length, which is an sRNA characteristic that enables the association of AGO2 and AGO4. Approximately 60.5% of the sRNAs that were 23 nt in length (associated with AGO5) had cytosine as their 5′ nucleotide. These results indicated that different classes of sRNAs in peanut exhibit different 5′ nucleotide biases, which is consistent with that in soybean [29].

### Identification of known and novel miRNAs in peanut embryos

To identify the miRNAs in the six sRNA libraries, all the unannotated reads that were 18–30 nt in length were compared with the plant miRNAs in miRBase (Release 21.0, July 2014). A total of 161 miRNAs, including 29 known miRNAs composing 19 miRNA families and 132 novel miRNAs, were identified (Additional file 9: Table S5). Of the 29 known miRNAs, 13 were members of 7 miRNA families conserved across various plant species. Sixteen miRNAs, including miR3509, miR3511, and miR3512, grouped into 12 peanut-specific families (Additional file 9: Table S5). miR159 and miR167 were most abundant in the conserved miRNA families, which is consistent with that in soybean [29]. Among the peanut-specific miRNAs, miR3514 and miR3518 were the most abundant (Additional file 9: Table S5). After the known miRNAs were identified, the remaining unique reads were used to identify the novel miRNAs; 132 novel miRNA candidates, named sequentially as ahy_novel_miRn1 to ahy_novel_miRn132, were identified (Additional file 9: Table S5). Quantitative real-time PCR (qRT-PCR) was performed to validate the novel miRNAs, and the predicted miRNAs were differentially expressed in peanut embryos under calcium deficiency and sufficiency conditions (Fig. 5).

### Calcium deficiency-responsive miRNAs in peanut embryos

**Fig. 4:**
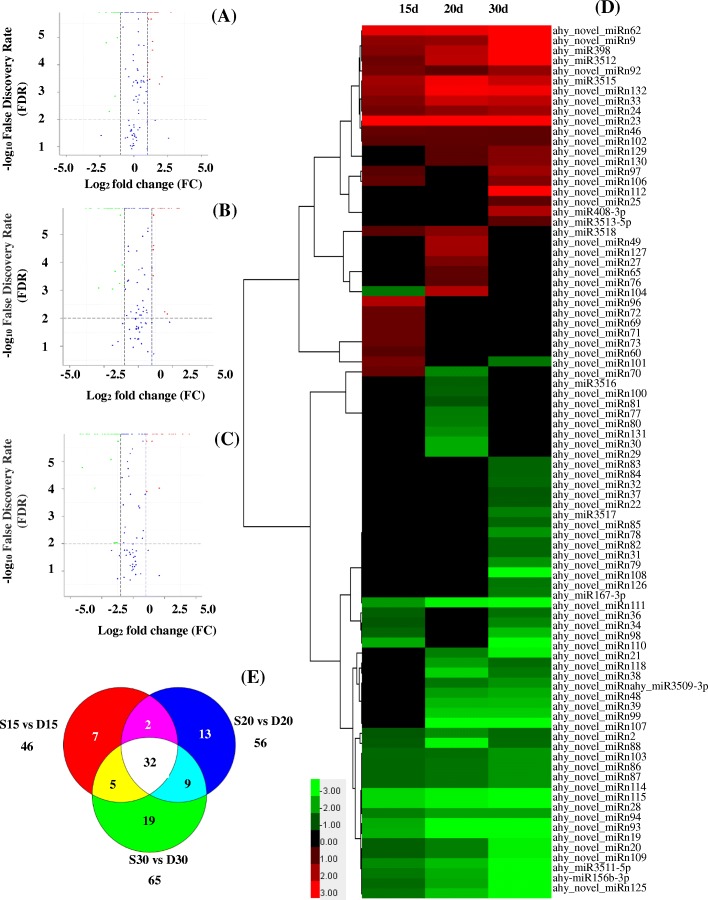
Differentially expressed miRNAs between libraries. **a** Differentially expressed miRNAs between S15 and D15. **b** Differentially expressed miRNAs between S20 and D20. **c** Differentially expressed miRNAs between S30 and D30. **d** Heatmap of differentially expressed miRNAs. **e** Distribution of differentially expressed miRNAs. *P* values were transformed to negative log_10_ values, and fold changes of miRNA expression between libraries were transformed to log_2_ values. Negative log_10_ P values and log_2_-fold changes are shown on the y- and x-axes, respectively. The green spots indicate downregulated miRNAs, the red spots indicate upregulated miRNAs, and the blue spots indicate miRNAs whose expression did not evidently change between the libraries. The data are presented as log_2_ (fold change) values comparing miRNA abundances (TPM) between D15 and S15, D20 and S20, and D30 and S30

To identify the miRNAs in peanut that respond to calcium deficiency, the normalized expression levels of the miRNAs in the six libraries were compared. The results showed that 87 miRNAs were differentially expressed under calcium deficiency and sufficiency during early embryo development (Fig. 4, Additional file 10: Table S6). Of these miRNAs, 12 were known miRNAs, and 75 were predicted to be novel miRNAs. Among these novel miRNAs, 32 were differentially expressed at three developmental stages, and 7, 13 and 19 miRNAs were differentially expressed at 15, 20 and 30 DAP, respectively (Fig. 4e). In addition, after the miRNA reads were normalized to transcripts per million (TPM), the expression of 23, 21 and 20 miRNAs was upregulated in response to calcium deficiency in D15, D20 and D30, respectively, and the expression of 23, 35 and 45 miRNAs was downregulated, respectively (Fig. 4). Cluster analysis of the differentially expressed miRNAs is illustrated in Fig. 4(D). The most upregulated miRNAs included ahy_novel_miRn112, ahy_novel_miRn23, ahy_novel_miRn62, ahy_novel_miRn132, ahy-miR3515, ahy-miR398, ahy-miR3512, and ahy_novel_miRn9. The most significantly downregulated miRNA was ahy_novel_miRn111, with a 29.06-fold change, while ahy_novel_miRn114, ahy_novel_miRn115, ahy_novel_miRn94 and ahy_novel_miRn93 were downregulated more than 3-fold (Additional file 10: Table S6).

**Fig. 5:**
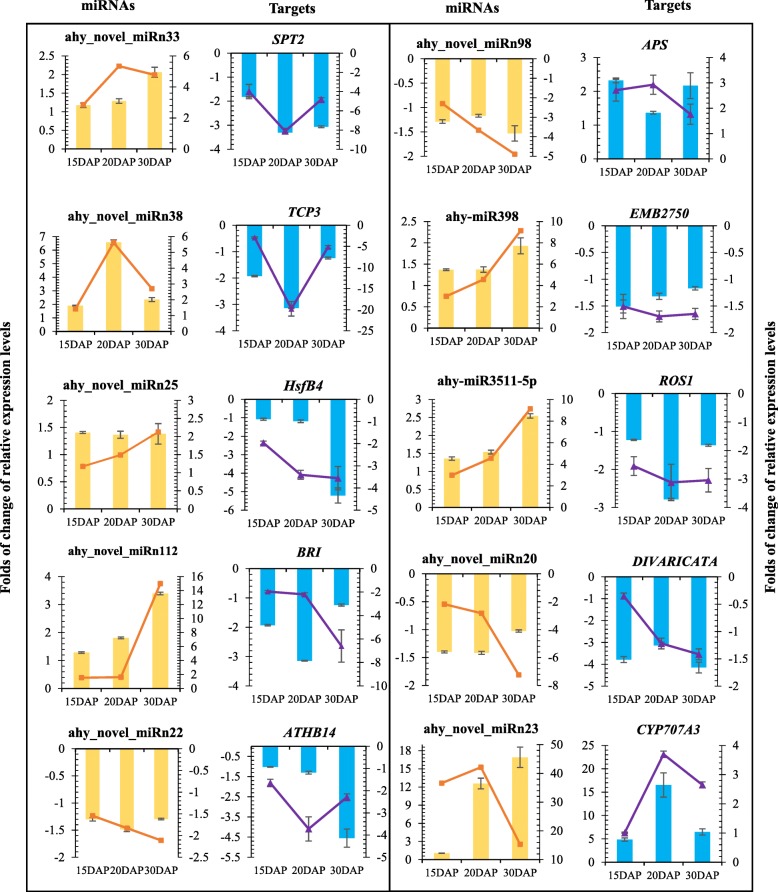
Quantitative RT-PCR validation of differentially expressed miRNAs and their corresponding target genes under calcium deficiency and sufficiency. The yellow bar represents relative changes in expression levels, as calculated by the 2^-△△CT^ method; qRT-PCR analysis was used to analyze the differentially expressed miRNAs. The orange line indicates relative changes in expression levels for differentially expressed miRNAs, as determined by RNA-seq. The blue bars represent changes in relative expression levels of target genes, as determined by qRT-PCR. The purple line indicates changes in relative expression levels of target genes, as calculated by the FPKM using RNA-seq. All qRT-PCRs and RNA-seq analyses were performed for three biological replicates

The expression levels of some calcium deficiency-responsive miRNAs were subsequently measured by qRT-PCR. The qRT-PCR results were consistent with that obtained by miRNA sequencing (Fig. 5). The differential abundance of different miRNAs between sufficient and deficient calcium levels in the soil suggested a possible miRNA-mediated regulation of gene expression during peanut embryo development.

### Target prediction

To elucidate the regulatory role of miRNAs during early peanut embryo development, the miRNA target genes were identified by TargetFinder software. A total of 197 targets were found for 161 miRNAs. Among these targets, 117 were characterized for 87 differentially expressed miRNAs. Most of the identified target genes were predicted to encode proteins involved in transcriptional regulation, protein modification, protein degradation and hormone synthesis. Consistent with the results of previous studies, most targets of the conserved miRNAs encoded transcription factors such as NAC, Myb, AP2, basic helix-loop-helix (bHLH)-MYC, Heat stress transcription factor B-4, TCP3, and DIVARICATA (Additional file 10: Table S6). Some target genes were annotated and classified as enzyme-coding genes (LRR receptor-like kinase, SRSF protein kinase, Serine/threonine protein kinase), resistance proteins (PCR8, disease resistance protein Rpp4C1, BTB/POZ domain-containing protein), proteins responsive to stresses (TPR repeat-containing thioredoxin TTL1, pentatricopeptide repeat-containing protein), proteins related to hormone metabolism (Cytochrome P450) and other proteins (Additional file 10: Table S6). Of the 132 novel miRNAs, 68 (51.5%) had putative targets in the annotated gene sets of *Arachis duranensis* and *Arachis ipaensis*.

### Kyoto encyclopedia of genes and genomes (KEGG) pathway analyses of the targets of differentially expressed miRNAs

For functional prediction, the targets of the identified differentially expressed miRNAs were subjected to different databases; a total of 186 targets (94.4%) were annotated. Our evidence demonstrated that the target genes were significantly enriched in signal transduction and cell communication and involved in various biological processes such as embryo development, pollen development and protein ubiquitination (Additional file 3: Figure S3). Cluster of Orthologous Groups of Proteins (COG) functional classification revealed that target genes that function in replication, recombination and repair, transcription, translation and signal transduction were detected at each stage of embryo development. Notably, targets that function in coenzyme transport and metabolism, posttranslational modification, protein turnover, chaperoning, inorganic ion transport and metabolism were detected in embryos at 20 DAP. Targets that function in RNA processing and modification, cell cycle control, cell division, chromosome partitioning, carbohydrate transport and metabolism were detected in embryos at 30 DAP (Additional file 4: Figure S4).

KEGG analysis revealed that 19 differentially expressed target genes were significantly enriched in 13 pathways, including plant hormone signal transduction, starch and sucrose metabolism, amino sugar and nucleotide sugar metabolism, and ubiquitin-mediated proteolysis (Additional file 11: Table S7). These findings highlighted the significant regulatory activity of miRNAs during peanut embryo development via involvement in plant hormone signal transduction, reserve metabolism and posttranslational modification.

### Correlation analyses between miRNAs and target mRNAs

**Fig. 6:**
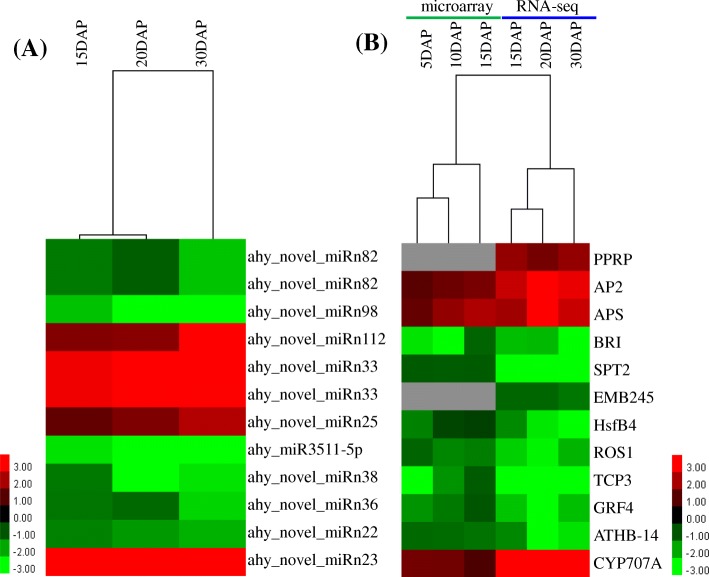
Combined view of the expression levels of differentially expressed miRNAs and their target genes. **a** The expression of differentially expressed miRNAs and (**b**) the expression of their corresponding target genes. The original expression values of miRNAs and their target genes are presented as log_2_-fold changes, which were determined by comparing miRNA abundances (TPM) between D15 and S15, D20 and S20, and D30 and S30

Integrated analysis of miRNAs and their target expression helps to reveal the regulatory pathways of miRNAs and identify functional miRNA-mRNA modules. To investigate the expression patterns of the global transcriptome of peanut embryos under low and high calcium levels, RNA-seq libraries for calcium deficiency and sufficiency at 15, 20 and 30 DAP were constructed and the global gene expression profiles surveyed using the Illumina HiSeq™ 2500 platform. Then the normalized expression levels of all genes were subsequently analyzed for their expression patterns to identify differentially expressed genes. Among these differentially expressed genes, a total of 52 target genes of 20 miRNAs in peanut embryos were differentially expressed under low and high calcium levels (Additional file 10: Table S6). Among them, only 8 and 12 miRNA-target pairs showed negative and positive correlation patterns, respectively (Fig. 6, Additional file 10: Table S6). For example, the expression of ahy_novel_miRn23 was significantly upregulated under low calcium levels, followed by a similar increase in the expression of its targets CYP707A1 and CYP707A3. To further understand the expression profiles of targets at early developmental stages (5, 10, 15 DAP) under calcium deficiency and sufficiency conditions, a microarray analysis of targets was performed for 12 target genes. Ten important genes including *AP2*, *APS, BRI1*, *SPT2*, *HsfB4*, *ROS1*, *TCP3*, *GRF4*, ATHB-14 and *CYP707A* showed different expression at 5,10 and 15DAP (Fig. 6, Additional file 10: Table S6). Interestingly, the expression of abscisic acid 8′-hydroxylase (CYP707A1 and CYP707A3) was upregulated in peanut embryos under calcium deficiency (Figs. 5 and 6). Abscisic acid 8′-hydroxylase is a key enzyme that negatively controls endogenous ABA levels. ABA is generally considered a phytohormone that inhibits growth and enhances adaptation to various stresses in plants [33]. Our results imply that moderate endogenous ABA levels possibly are vital for peanut embryo development. The expression of pentatricopeptide repeat-containing protein (PPRP), which is involved in RNA modification, was upregulated under calcium deficiency (Fig. 6). The expression of the floral homeotic protein APETALA 2 (AP2), which is related to flower, ovule and seed development, also increased under calcium deficiency (Figs. 5 and 6). Among the downregulated target genes, the expression of several transcription factors of the growth-regulating factor (GRF) family, including GRF3, 4, 5, 6, and 9, was downregulated (Fig. 6). The OsmiR396c-OsGRF4-OsGIF1 regulatory module was reported to play important roles in the determination of grain size and yield in rice [34]. As a member of the TCP family, TCP3 might participate in embryogenesis [35]. However, the expression of TCP3 was downregulated in aborted peanut embryos (Figs. 5 and 6). The expression of two other cell division- and proliferation-related transcription factors, HsfB4 and DIVARICATA, also decreased under calcium deficiency (Figs. 5 and 6). The expression of Brassinosteroid-Insensitive 1 (BR1), which perceives brassinosteroids (BRs) and initiates BR signaling, was similarly downregulated (Figs. 5 and 6), implying that BRs play important roles in peanut embryo development. Taken together, these results suggest a direct miRNA-target expression modulation in peanut embryo development under calcium deficiency conditions.

**Fig. 7:**
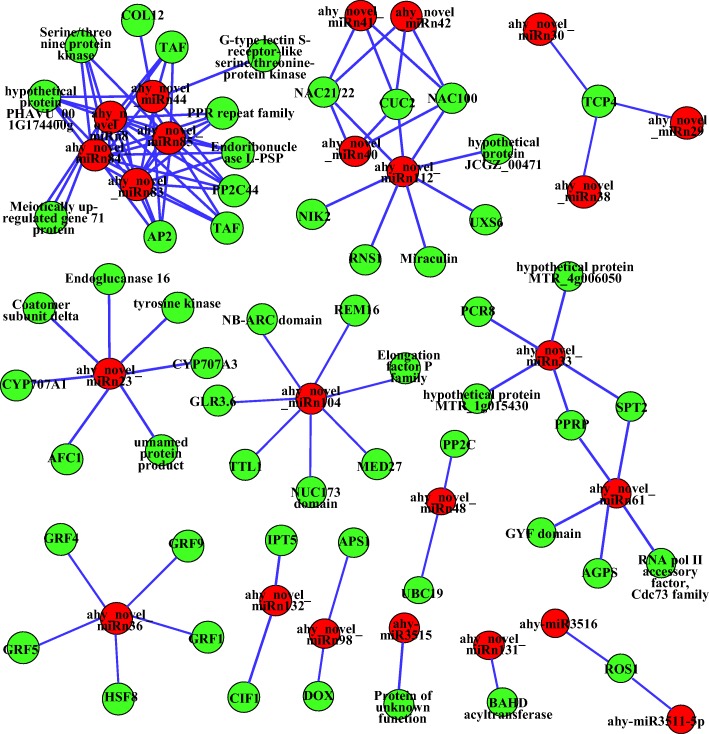
miRNA-mediated regulatory network constructed by Cytoscape (version 3.4.0). The red and green circles represent the miRNAs and their targets, respectively

Further qRT-qPCR analysis validated the expression profiles of ten interesting miRNA-target modules (Fig. 5). These results suggested that miRNAs significantly modulate their target mRNA accumulation at the posttranscriptional level to the appropriate expression level for controlling early peanut embryo development. Several differentially expressed miRNA-target regulatory networks were constructed accordingly (Fig. 7). The differentially expressed miRNAs and targets formed miRNA-target pairs whose expression was negatively or positively correlated during embryo development. Taken together, these findings suggest that differentially expressed miRNAs certainly play fundamental regulatory roles in various aspects of biological processes during peanut embryo development.

## Discussion

Increasing amounts of evidence have indicated that miRNAs regulate plant seed formation and development [18]. Peanut embryo development is a complex process that involves the activity of a series of gene regulatory pathways at both the transcriptional and posttranscriptional levels. A number of miRNAs related to peanut growth have been identified [25, 26]. Moreover, previous work has documented the importance of calcium in peanut development [1, 2]. However, the involvement of miRNA regulation in peanut embryo abortion under calcium deficiency conditions has not been elucidated. In this study, based on the phenotypes of peanut pods under calcium deficiency and sufficiency conditions, certain miRNAs were differentially expressed in peanut embryos under calcium deficiency and sufficiency conditions, and their related target genes were predicted to control peanut embryo development. Integrated analysis of miRNAs and transcriptome expression and microarray analysis revealed potential miRNA-mRNA modules that are probably related to peanut embryo responses to calcium deficiency conditions. Notably, this study is the first to investigate miRNA regulatory mechanisms involved in peanut embryo development in response to calcium deficiency.

### miRNA expression profiles significantly differed between peanut embryos under low and high calcium conditions

sRNAs regulate gene expression posttranscriptionally in plants and animals. Identifying miRNAs and their functional modules is important for clarifying the mechanisms that underlie plant growth and development. Deep sequencing strategies represent powerful technologies for discovering miRNAs and profiling their expression, and these strategies have been applied to many plant species, such as soybean [29], cotton [36] and peanut [25]. Calcium deficiency in the soil induces early embryo abortion in peanut, resulting in the production of empty pods [4]. Although a number of miRNAs have been identified in peanut [25, 26, 30, 37, 38], embryo abortion under calcium deficiency conditions and the involvement of miRNAs have not been investigated. To dissect the miRNA-mediated regulatory network of embryo abortion under calcium deficiency, sRNA libraries were constructed using calcium deficiency- and sufficiency-treated peanut embryos. The libraries were sequenced via a high-throughput sequencing strategy. The results showed that calcium deficiency caused significant variation in the expression of miRNAs in early peanut embryos. A total of 161 miRNAs were identified. Notably, the expression levels of 87 miRNAs were significantly affected by calcium deficiency, with the majority downregulated.

miR408 was identified to play a vital role in iron (Fe) uptake [39], affecting copper levels in cells (Abdel-Ghany and Pilon, 2008) and responding to drought [16, 40]. In wheat, miR408 functions in heading time by mediating the expression of *TaTOC1*s [41]. miR408 is significantly involved in various abiotic stress responses and plays a central function in plant survival [42–44]. In this study, the expression of ahy-miR408 was significantly upregulated in the embryos at 30 DAP. This upregulation of ahy-miR408 might modulate a series of target genes that affect the normal development of peanut embryos. miR398 can be suppressed by carbon (C), nitrogen (N), and sulfur (S) deficiency in *Arabidopsis* [45]. In addition, miR398 is also responsive to deficiencies in other nutrient elements, including cadmium [46], copper [47], zinc [48] and phosphate [49]. The expression of miR398 is rapidly induced by heat stress, and miR398 is critical for thermotolerance in *Arabidopsi*s [50]. In addition, miR398 expression is significantly upregulated in imbibed seed in maize [51]. Here, we also observed that the expression of ahy-miR398 was upregulated in peanut embryos under calcium deficiency, which is different from previous results concerning C, N and S stress but similar to Cu deficiency [47]. Plants absorb sufficient amounts of nutrients to maintain normal growth and development, but different nutrients involve interdependent metabolic pathways [52]. A certain nutrient deficiency could specifically induce miRNAs to mediate target mRNA expression to maintain the balance of different nutrients [45]. Interestingly, miR398 and miR408 are coexpressed in response to many stresses. For example, the expression of both miR398 and miR408 is upregulated in response to water deficit in *Medicago truncatula* [53] but downregulated in pea [54]. Both miRNAs were proposed to be related to copper homeostasis in *Arabidopsis* [55, 56]. In the present study, the expression of both miR398 and miR408 was upregulated, indicating their roles in embryo abortion in peanut under calcium deficiency. miR167 targeting of ARF6 and ARF8 is essential for the fertility of ovules and anthers in *Arabidopsis* [57]. A recent report demonstrated that the expression of miR167 is essential for regulating gynoecium and stamen development in immature tomato flowers, as this miRNA modulates the expression levels of *SlARF6* and *SlARF8* [58]; moreover, miR167 expression in asparagus was shown to be significantly greater in female plants than in male plants [59]. The expression of both miR167 and miR156 was downregulated under Fe deficiency in the roots and shoots of high-Fe rice lines [60]. The current results indicate that the expression of both miR167 and miR156 was downregulated under calcium deficiency in peanut embryos. Together, these results suggested that calcium deficiency-responsive miRNAs could be involved in peanut embryo abortion.

### Differentially expressed miRNAs regulate embryo development by mediating target mRNAs

#### Transcription factor genes

The majority of the targets of the differentially expressed miRNAs that might be involved in peanut embryo abortion were predicted to encode transcription factors. Numerous studies have demonstrated that the expression of specific transcription factors is mediated by miRNAs during plant embryo development. For example, miR167 regulates ARF6 and ARF8 in *Arabidopsis* [61]; miR160a regulates ARF10, ARF16 and ARF17 [62, 63]; miR166 regulates class III homeodomain leucine zipper (HD-ZIP III) transcription factors [64]; and miR172 regulates AP2 transcription factors [37, 65]. In the current study, the most significant expression difference occurred for ahy_novel_miRn112 in the embryos under calcium deficiency at 30 DAP. Interestingly, the targets of ahy_novel_miRn112 were NO APICAL MERISTEM and CUP-SHAPED COTYLEDON (NAM/CUC), as well as NAC, NAD and CUC2, which play important roles in mediating the gene expression response to auxin and regulating ovule development. Therefore, the increased abundance of ahy_novel_miRn112 might partly reduce the expression of NAC and CUC2, ultimately resulting in abnormal embryo development under calcium deficiency. Expression of ahy_novel_miRn129 and ahy_novel_miRn130, which target the transcription factor LONESOME HIGHWAY (LHW), was upregulated under calcium deficiency. LHW encodes a bHLH transcription factor and was indicated to be a key regulator that initiates vascular cell differentiation in association with auxin regulation [66]. By forming TARGET OF MONOPTEROS5 (TMO5)/LHW bHLH heterodimers, LHW is required for embryonic vascular tissue establishment in and indeterminate growth of *Arabidopsis* during the first division of vascular cells in embryos [67]. ahy_novel_miRn25 expression was also upregulated under calcium deficiency. The accumulation of miR159 results in delayed heading time and male sterility in rice [68]. The target of ahy_novel_miRn25 is the transcription factor gibberellic acid MYB (GAMYB), which is expressed in response to GA signal transduction. GAMYB was reported to be regulated by miR159 [69, 70]. GAMYB expression promotes programmed cell death in seeds (aleurone) and anthers (tapetum) [71]. A growing body of evidence has demonstrated that GAMYB is involved in many aspects of plant growth and development, including anther development [72], floral initiation [73], sex differentiation [74], seed development [75] and seed germination [76]. Ahy_novel_miRn25 was predicted to target heat stress transcription factor B-4 (HsfB4), which was reported to regulate the asymmetry of stem cell division in *Arabidopsis* [77]. The expression of ahy_novel_miRn30, ahy_novel_miRn29 and ahy_novel_miRn38 was downregulated, and their target was the transcription factor TEOSINTE BRANCHED1, CYCLOIDEA, PROLIFERATING CELL FACTORS 4 (TCP4), which is involved in jasmonic acid (JA) biosynthesis [78, 79]. TCP4 is posttranscriptionally regulated by miR319 and plays key roles during cell proliferation to cell expansion and differentiation [80]. Recently, TCP4 was reported to control petal size and shape [81], pollen germination [79] and hypocotyl elongation [82] in *Arabidopsis*. As a member of the TCP family, TCP3 plays an important role in embryogenesis [35]. Here, the expression of TCP3 was downregulated in aborted peanut embryos, and recent reports have shown that the expression of TCP3 is upregulated in enlarged peanut embryos [35]. Among the downregulated target genes, the expression of several GRF family transcription factors, including GRF3, 4, 5, 6, and 9, was downregulated. GRFs have been reported to play important roles in seed formation [83, 84]. In addition, the OsmiR396c-OsGRF4-OsGIF1 regulatory module has been reported to play important roles in the determination of rice grain size and yield [34].

The expression of ahy_novel_miRn1 and ahy_novel_miRn20 was downregulated under calcium deficiency, and the target of these miRNAs was the transcription factor DIVARICATA. DIVARICATA is involved in floral symmetry and cell proliferation during the early stages of pollen development [85]. The decreased abundance of ahy_novel_miRn19 and ahy_novel_miRn20 under calcium deficiency could partly reduce the expression of DIVARICATA, ultimately influencing embryo development. In summary, a number of transcription factors (targets of miRNAs) were differentially expressed in peanut embryos under calcium deficiency, suggesting that the miRNA-mediated regulation of these transcription factors might play crucial roles in peanut embryo abortion under calcium deficiency.

#### Phytohormone homeostasis-related genes

Phytohormone homeostasis is extremely important for plant growth and development. ABA has been reported to play important roles in response to various stresses, especially drought. In this study, the results showed that ahy_novel_miRn23, whose expression was significantly upregulated under calcium deficiency, targets *CYP707A1* and *CYP707A3*. According to the transcriptome sequencing, the expression of *CYP707A1* and *CYP707A3* was significantly upregulated under calcium deficiency. Both *CYP707A1* and *CYP707A3* belong to the cytochrome P450 family and encode ABA 8′-hydroxylase, which converts ABA to phaseic acid (PA) and subsequently to 4′-dihydrophaseic acid (DPA) [86, 87]. In *Arabidopsis,* the CYP707A family comprises four genes (*CYP707A1*, *CYP707A2*, *CYP707A3* and *CYP707A4*). Endogenous ABA levels have been reported to be a positive regulator during plant embryo development [88]. The exogenous spraying of ABA may be effective at increasing Ca^2+^ concentrations in tomato [89, 90] and apple [91]. Overexpression of *PvCYP707A1*, *PvCYP707A2* and *PvCYP707A3* in *Nicotiana sylvestris* resulted in wilted phenotypes with reduced ABA levels but increased PA levels, which demonstrated that the expression of *PvCYP707A*s was the major regulatory factor for ABA catabolism in bean [92]. *CYP707A4* was isolated from peanut in this laboratory, and the overexpression of *AhCYP707A4* in *Nicotiana benthamiana* resulted in phenotypes with low ABA contents but an abundance of aborted embryos, small pods and fewer numbers of seeds. Therefore, *CYP707A4* could be a key player in the regulation of Ca^2+^ deficiency-induced embryo abortion via ABA-mediated apoptosis in embryo abortion [4]. It is therefore predicted that both CYP707A1 and CYP707A3 play important roles in peanut embryo development.

BRs may play key roles in plant development [93]. BR1 is a receptor for BRs and can perceive BRs and initiate BR signaling. In our study, the expression of BR1 was downregulated in aborted embryos. In tomato, SIBR1 overexpression increased fruit numbers and yield [94]. Here, BR1 downregulation might affect the BR signaling pathway, leading to embryo abortion. Further investigation is required for a detailed mechanism of this possibility.

#### Posttranslational modification-related genes

Ubiquitination is a eukaryotic posttranslational protein modification mediated by three classes of enzymes, E1 (a ubiquitin-activating enzyme), E2 (a ubiquitin-conjugating enzyme) and E3 (a ubiquitin-ligase enzyme), and is involved in regulating numerous biological processes. In this study, the expression of two miRNAs (ahy_novel_miRn103 and ahy_novel_miRn48) targeting the E3 ubiquitin-protein ligase and the ubiquitin-conjugating enzyme E2 (UBC19) was downregulated. UBC19 and UBC20 participate specifically in cyclin B1 degradation and play key roles during the cell cycle [95]. UBC19 and UBC20 might also be involved in ubiquitination during differentiation and/or in differentiated cells [95]. E3 ubiquitin ligases interact with specific degradation substrates for ubiquitination and often proteolytic degradation via the 26S proteasome. In *Arabidopsis*, there are seven members of HECT E3 ligases named UPL1 to UPL7, and they are probably one of the least diverse classes of known plant E3 ligases [96]. Unfortunately, the biological functions of these E3 ligases were unclear. Our results suggest that peanut embryo abortion under calcium deficiency might be caused by posttranslational modification.

#### Other important genes

Expression of the ahy_novel_miRn9, ahy_novel_miRn33 and ahy-miR398 genes was upregulated under calcium deficiency. The predicted targets of these miRNAs encode putative pentatricopeptide repeat-containing proteins (PPRs), which have been demonstrated to play important roles in the first mitotic division during gametogenesis and in cell proliferation during embryogenesis [97]. The increased abundance of these three miRNAs then reduces the expression of PPRPs, subsequently leading to embryo abortion. Among the downregulated miRNAs, ahy_novel_miRn111 was the most downregulated. Expression of the ahy_novel_miRn93, ahy_novel_miRn94, ahy_novel_miRn114, ahy_novel_miRn115, ahy_novel_miRn125, ahy_novel_miRn107 and ahy_novel_miRn109 genes was downregulated by more than 3-fold. However, the targets of these downregulated miRNAs were not identified. Nevertheless, expression of the ahy-miR156b-3p and ahy-miR3511-5p genes was downregulated. The target of ahy-miR3511-5p was ROS1, which encodes a DNA glycosylase/lyase, a repressor of transcriptional gene silencing in *Arabidopsis* [98].

Other targets are also expected to have broad effects on embryo development because they are predicted to play roles in the biosynthesis of plant hormones (e.g., cytokinin, ABA, auxin), plant–pathogen interactions (e.g., receptor kinases) and signal transduction (e.g., endoglucanase, protein phosphatase 2C). Furthermore, notably, no calcium signaling pathway-related genes were predicted in this study. It is possible that the regulation of miRNA-mediated posttranscriptional levels might be initiated after calcium signaling transduction for peanut embryo abortion under calcium deficiency conditions.

### miRNA-mediated regulatory network of peanut embryo abortion under calcium deficiency

According to the correlations between differentially expressed miRNAs and their targets, a schematic model was proposed for the miRNA-mediated regulatory network of embryo abortion during embryo development under calcium deficiency in peanut (Fig. 7). Targets of these differentially expressed miRNAs contain important transcription factors and functional proteins involved in various biological processes (Fig. 6). The expression of the miRNAs that target genes related to plant hormone biosynthesis and signal transduction, starch and sucrose metabolism, and organelle regulation was upregulated. In addition, the expression of miRNAs targeting the repression of cell proliferation, autophagy, posttranslational modification, proteolysis, floral organ development and plant defense responses was upregulated.

Taken together, our results pave an important avenue for unraveling the complex miRNA-mediated regulatory network during embryo development in peanut under calcium deficiency.

## Conclusions

miRNA sequencing together with transcriptome profiling and gene chip analysis were performed to reveal the miRNA-mediated regulation of peanut embryo abortion under calcium deficiency. A total of 29 known and 132 potential novel miRNAs composing 12 peanut-specific miRNA families were discovered. Among these novel miRNAs, 87 were differentially expressed during early embryo development under calcium deficiency and sufficiency conditions, and 117 of their target genes were identified. Integrated miRNA and transcriptome analysis and gene chip expression analysis resulted in the identification of 52 differentially expressed target genes of 20 miRNAs. These differentially expressed miRNAs and their corresponding target genes probably play key roles in the regulation of peanut embryo abortion under calcium deficiency. These findings provide for the first time new insights into miRNA-mediated regulatory pathways involved in peanut embryo abortion under calcium deficiency.

## Methods

### Plant materials and growth conditions

Baisha1016, which is a popular cultivated peanut variety in China and has been preserved in our lab, was used as plant material and grown in Ca^2+^-deficient soil in Pingtan, Fujian Province, China. The exchangeable Ca^2+^ content in the soil was 0.6 cmol/kg soil. The peanut plants grown in this soil were used for Ca^2+^-deficiency experiments, and those grown in identical soil fertilized with 75 kg 667/m^2^ plaster were used for Ca^2+^-sufficiency experiments. The exchangeable Ca^2+^ content in the soil after fertilization was 4.2 cmol/kg soil. The critical Ca^2+^ content in the soil that induced peanut embryo abortion was generally < 3.0 cmol/kg soil. The embryos were classified based on their developmental stage and visual morphology. Embryos (15, 20 and 30 DAP) were manually dissected, frozen in liquid N and then stored at − 80 °C for subsequent experiments. Three biological replicates were prepared for each treatment. The samples were named S15, S20 and S30 (15, 20, 30 DAP under calcium sufficiency, respectively) as well as D15, D20 and D30 (15, 20, 30 DAP under calcium deficiency, respectively).

### sRNA library construction and sequencing

Total RNA was isolated using TRIzol reagent according to the manufacturer’s instructions (Invitrogen, CA). A NanoPhotometer spectrophotometer (Implen, CA), a Qubit RNA Assay Kit and Qubit 2.0 Fluorometer (Life Technologies, CA) and an RNA Nano 6000 Assay Kit in conjunction with ab Agilent Bioanalyzer 2100 system (Agilent Technologies, CA) were used to detect the purity, concentration and integrity of the RNA samples, respectively. Six sRNA libraries (S15, S20, S30, D15, D20, D30) were generated. For each library, 3 RNA samples from three biological replicates were pooled equally. The RNAs were pooled together and then used for sRNA library construction via a Next Ultra sRNA Sample Library Prep Kit for Illumina (NEB, Beijing, China). The sRNA libraries were then sequenced via the Illumina HiSeq 2500 platform (Biomarker, China).

### Bioinformatics analysis of sRNA sequencing

Raw reads were processed by in-house Perl scripts to remove adapter sequences, low-quality reads and repetitive reads. Reads smaller than 18 nt or longer than 30 nt were also removed. Moreover, the Q30 and GC contents were calculated accordingly. The clean reads were then aligned with the reference genome (http://www.peanutbase.org/home) [99] using Bowtie software (version 1.0.0) [100] for miRNA identification. No mismatches between sRNAs and the genome sequence were allowed. The reads that matched to rRNA, tRNA snRNA, snoRNA, protein-coding genes and other noncoding RNA (ncRNA) as well as repeats were subsequently excluded using Bowtie software (version 1.0.0, −v 0) [100]. The matched reads were aligned against known miRNAs in miRBase (http://www.mirbase.org, release 21.0) for known miRNA identification to avoid mismatch using miRDeep2 software [101]. For novel miRNA identification, the corresponding precursor sequences were checked with MIREAP (https://sourceforge.net/projects/mireap/) to confirm the precursors of the expected secondary structures. For the conserved miRNAs, the same miRNA/family names as those in miRBase were assigned but with new serial numbers (such as b, c) in some cases. With respect to the novel miRNAs, the names ahy_novel_miRn1 to ahy_novel_miRn132 were sequentially given.

The expression of miRNAs was normalized to TPM. The differential expression of the miRNAs was analyzed using the DESeq package (version 1.18.0, http://www.bioconductor.org/packages/release/bioc/html/DESeq.html) with the following criteria: |log_2_ fold change| ≥ 1 and a false discovery rate (FDR) ≤0.01 [102].

### Prediction and annotation of potential miRNA targets

The potential targets of the identified miRNAs in peanut were predicted via TargetFinder software (version 1.6, −c 3) [103]. The predicted targets of the identified miRNAs were subjected to nonredundant (NR), Swiss-Prot, Gene Ontology (GO), and COG analyses to predict their biological functions. In addition, the targets were compared with the KEGG, KOG and Pfam databases to determine their biological roles. KOBAS software was used to analyze the differentially expressed genes in the KEGG pathways. Based on the differentially expressed miRNAs and their corresponding targets, a miRNA-target regulatory network was constructed using Cytoscape software (version 3.4.0) [104].

### Transcriptome dataset for expression analysis of targets genes

Total RNA was extracted from peanut embryos under calcium deficiency or sufficiency using TRIzol reagent (Invitrogen, Carlsbad, CA). cDNA libraries were prepared using an Illumina Paired End Sample Prep Kit with three biological replicates and were sequenced on an Illumina HiSeq™ 2500 platform (Biomarker, China). After the raw reads were filtered to remove adapter sequences and low-quality reads, the remaining clean reads were aligned to the reference genome (http://www.peanutbase.org/home) [99] using Bowtie software (version 1.0.0) [100] and TopHat2 [105] for mapping locations. The mapped reads were subsequently assembled by Cufflinks (http://cufflinks.cbcb.umd.edu/) [106]. Fragments per kilobase of exon per million fragments (FPKM) were used to measure transcript sufficiency, which were used for targets genes expression analysis.

### qRT-PCR validation of miRNAs and mRNA targets

With respect to differentially expressed miRNAs, the quantification of mature miRNA abundance was examined using a Mir-X™ miRNA First Strand Synthesis Kit and a Mir-X™ miRNA qRT-PCR SYBR® Kit (Clontech, CA). Briefly, total RNA was extracted using TRIzol reagent (Invitrogen, CA) according to the manufacturer’s instructions. Genomic DNA (gDNA) was removed from the purified RNA using DNase I (Takara, Dalian, China) in accordance with the manufacturer’s instructions. Two micrograms of gDNA-free RNA was reverse-transcribed using the Mir-X™ miRNA First Strand Synthesis Kit (Clontech, CA). qRT-PCR was then performed using the Mir-X™ miRNA qRT-PCR SYBR® Kit (Clontech, CA). U6 was used as a reference gene for normalization. Regarding target genes, real-time PCR was performed to determine relative expression levels using ChamQ SYBR qPCR Master Mix (High ROX Premixed) (Vazyme, Nanjing, China). All reactions were performed on an Applied Biosystems ABI 7500 system (ABI, CA, USA) for three biological replicates for both miRNA and target mRNA analyses. The relative expression levels of the target genes were calculated using the comparative threshold cycle (CT) method (2^-△△CT^ method) [107] by normalizing the PCR threshold cycle number (Ct value) of the target gene to that of the reference gene *Ahactin*. Student’s t-test was subsequently used to compare differences between the control and experimental values. The primers used in all qRT-PCR experiments are listed in Additional file 12: Table S8.

### Microarray analysis of targets

To further understand the expression profiles of targets at early developmental stages (5, 10, 15 DAP) under calcium deficiency and sufficiency conditions, a microarray analysis of targets was performed. The microarray was designed as described previously, and the hybridization, washing, scanning and data analysis were also performed in accordance with previous methods [4]. The gene expression intensity of all hybridizations was analyzed, and the expression levels were estimated under calcium deficiency and sufficiency conditions. The expression data of the targets were normalized using quantile normalization [108] and generated using the robust multichip average algorithm [109]. Three replicates were performed for all experiments.

## Additional files

Additional file 1: **Figure S1.** Correlation coefficients of the samples. (DOCX 403 kb)

Additional file 2: **Figure S2.** miRNA nucleotide bias at the first position and each position. (DOCX 181 kb)

Additional file 3: **Figure S3.** GO enrichment of the targets of expressed miRNAs. (DOCX 4437 kb)

Additional file 4: **Figure S4.** COG functional classification of the target genes of differentially expressed miRNAs. (DOCX 2622 kb)

Additional file 5: **Table S1.** Summary of data from the sequencing of peanut sRNA libraries. (XLSX 13 kb)

Additional file 6: **Table S2.** Mapping results of sRNA sequences and the peanut genome. (XLSX 10 kb)

Additional file 7: **Table S3.** Size distribution of sRNA sequences. (XLSX 13 kb)

Additional file 8: **Table S4.** Distribution of the identity of the first nucleotide of different lengths of novel sRNAs. (XLSX 11 kb)

Additional file 9: **Table S5.** Summary of miRNA candidates identified in peanut. (XLSX 27 kb)

Additional file 10: **Table S6.** Differentially expressed miRNA and target gene expression in peanut embryos. (XLSX 37 kb)

Additional file 11: **Table S7.** KEGG pathways of the targets of differentially expressed miRNAs. (XLSX 12 kb)

Additional file 12: **Table S8.** Specific primer sequences used for qRT-PCR. (XLSX 10 kb)

## Abbreviations

ABA: Abscisic acid
AP2: APETALA 2
BR: Brassinosteroid
BR1: Brassinosteroid-Insensitive 1
Ca^2+^: Calcium
COG: Cluster of Orthologous Groups of Proteins
CYP707A1: Abscisic acid 8′-hydroxylase 1
CYP707A3: Abscisic acid 8′-hydroxylase 3
D15: 15 DAP under calcium deficiency
D20: 20 DAP under calcium deficiency
D30: 30 DAP under calcium deficiency
DAP: Days after pegging
DDRT-PCR: Differential display reverse transcription PCR
DPA: 4′-dihydrophaseic acid
FDR: False discovery rate
Fe: Iron
GA: Gibberellin
gDNA: Genomic DNA
GO: Gene Ontology
GRFs: Growth-regulating factors
HD-ZIP III: Class III homeodomain leucine zipper
HsfB4: Heat stress transcription factor B-4
KEGG: Kyoto Encyclopedia of Genes and Genomes
LHW: Transcription factor LONESOME HIGHWAY
miRNA: microrna
mRNA: messenger RNA
NAM/CUC: NO APICAL MERISTEM and CUP-SHAPED COTYLEDON
nt: nucleotide
PA: Phaseic acid
PPRP: Pentatricopeptide repeat-containing protein
qRT-PCR: Quantitative real-time PCR
rRNA: Ribosomal RNA
S15: 15 DAP under calcium sufficiency
S20: 20 DAP under calcium sufficiency
S30: 30 DAP under calcium sufficiency
snoRNA: Small nucleolar RNA
snRNA: Small nuclear RNA
SSHaLL: SSH-associated library lift
TCP: TEOSINTE BRANCHED1, CYCLOIDEA, PROLIFERATING CELL FACTORS
TPM: Transcripts per million
tRNA: Transfer RNA
