# Supplementary figures and images for: Integrated microRNA and transcriptome profiling reveals a miRNA-mediated regulatory network of embryo abortion under calcium deficiency in peanut (Arachis hypogaea L.)
Source: BMC Genomics. 2019 May 21;20:392. doi: 10.1186/s12864-019-5770-6 (PMC6528327; doi:10.1186/s12864-019-5770-6)

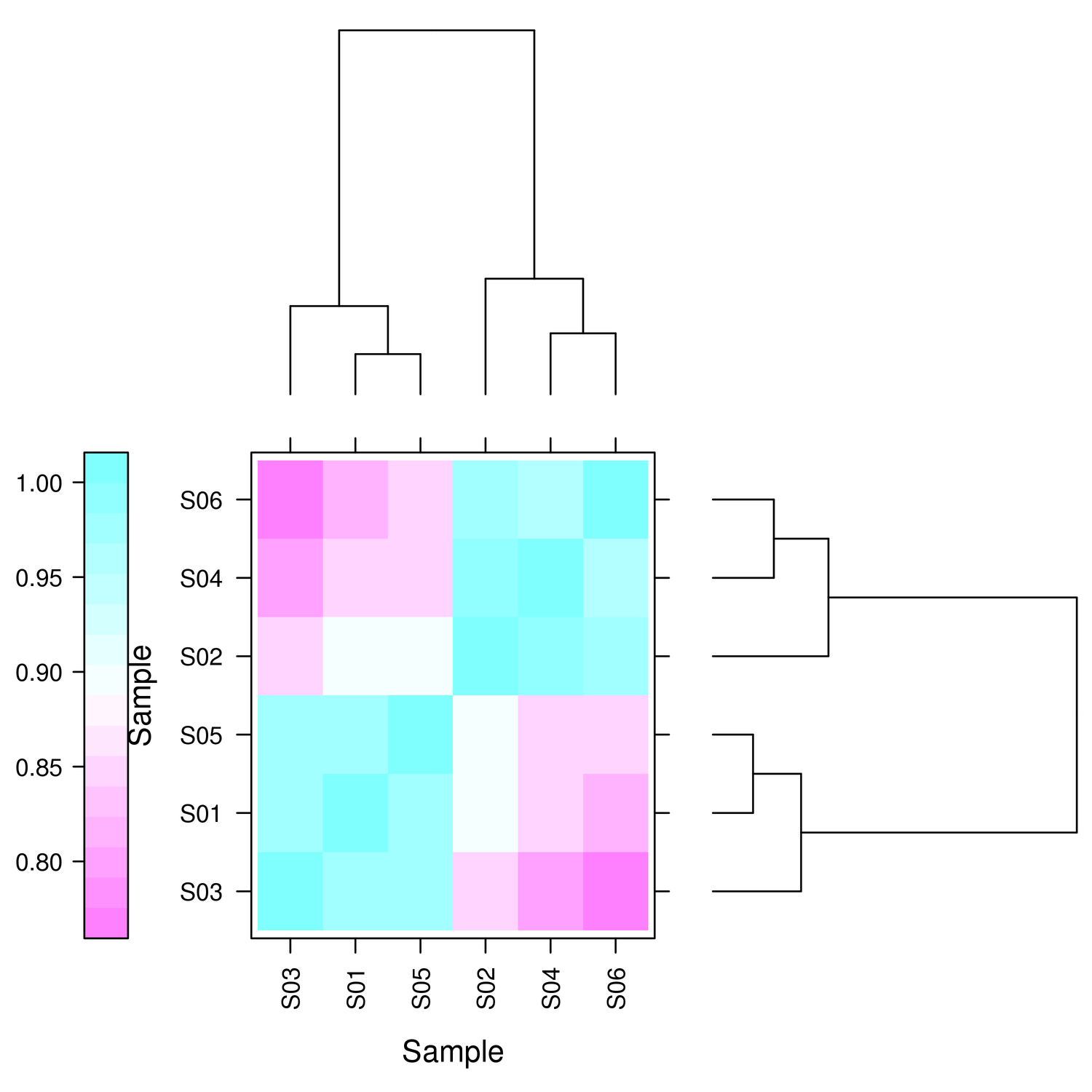


Samples


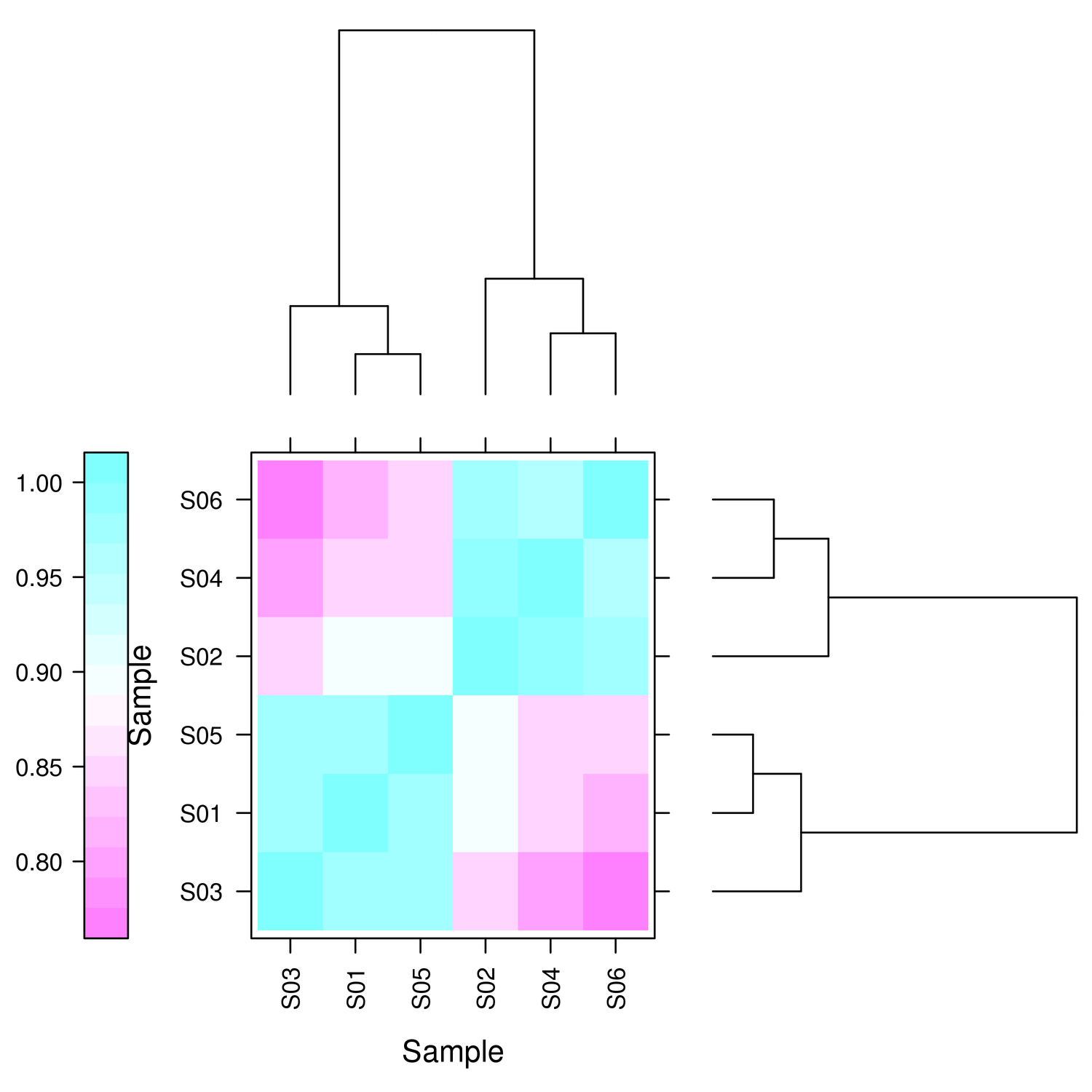


**D30**

**D20**

**D15**

**S30**

**S15**

**S20**

**S20 S15 S30 D15 D20** D30

Samples

Figure S1

Supplement: Supplementary file 1 — Figure S1. Correlation coefficients of the samples. (DOCX 403 kb) [file 12864_2019_5770_MOESM1_ESM.docx]

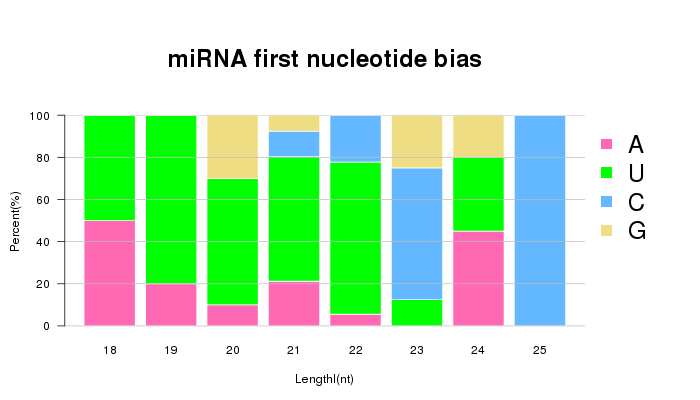

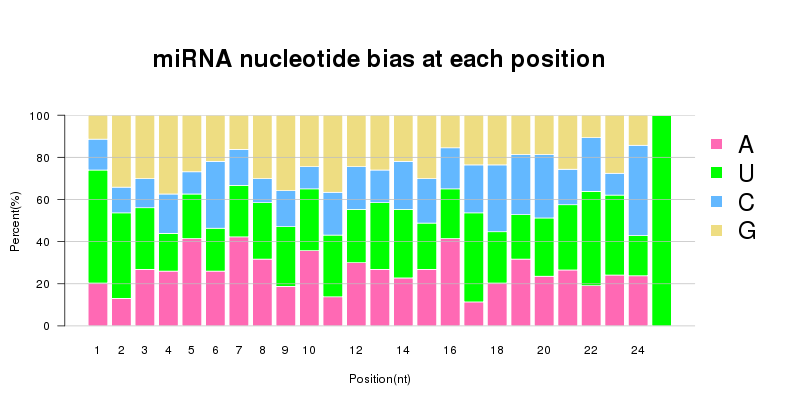


Figure S2

Supplement: Supplementary file 2 — Figure S2. miRNA nucleotide bias at the first position and each position. (DOCX 181 kb) [file 12864_2019_5770_MOESM2_ESM.docx]

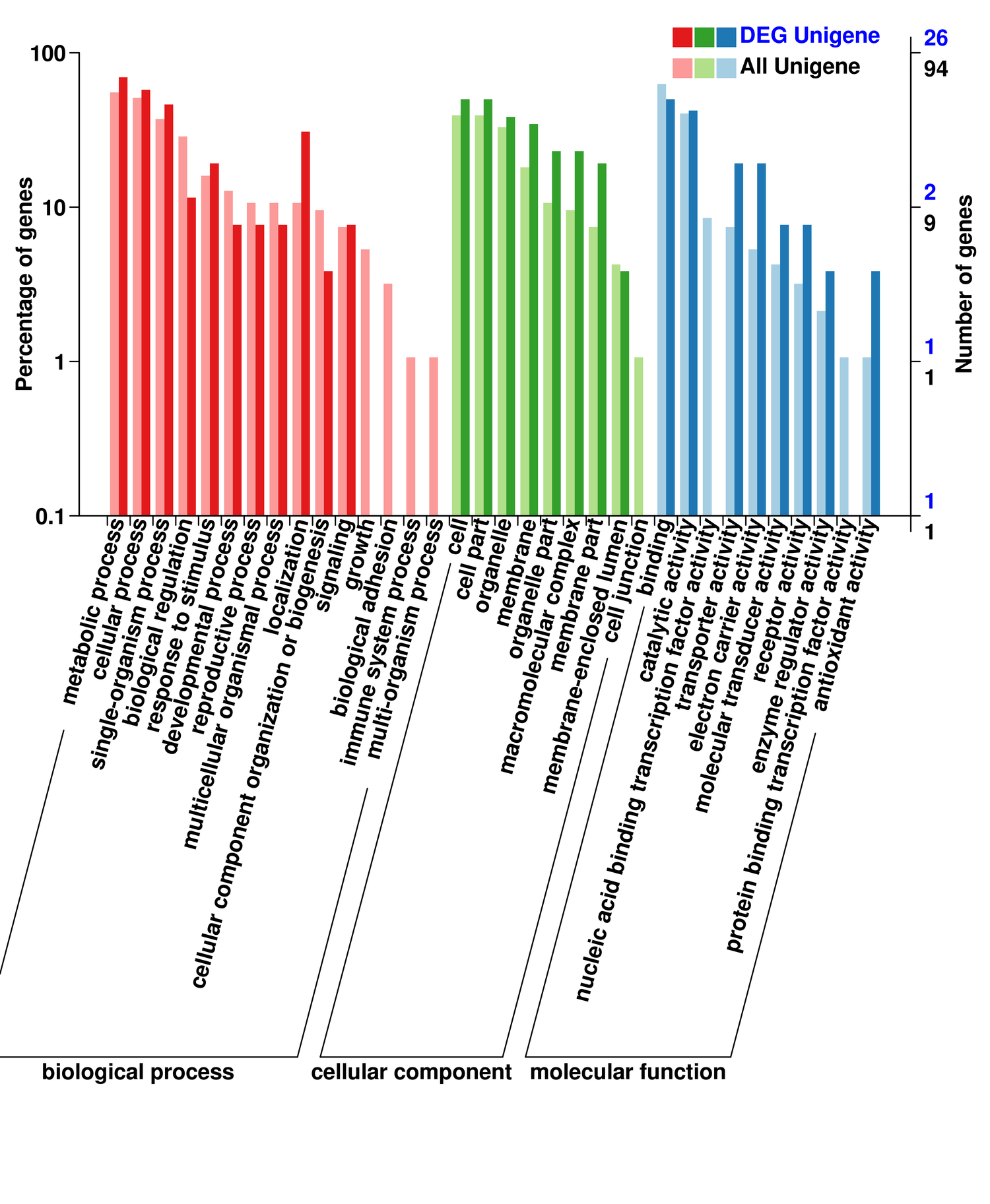

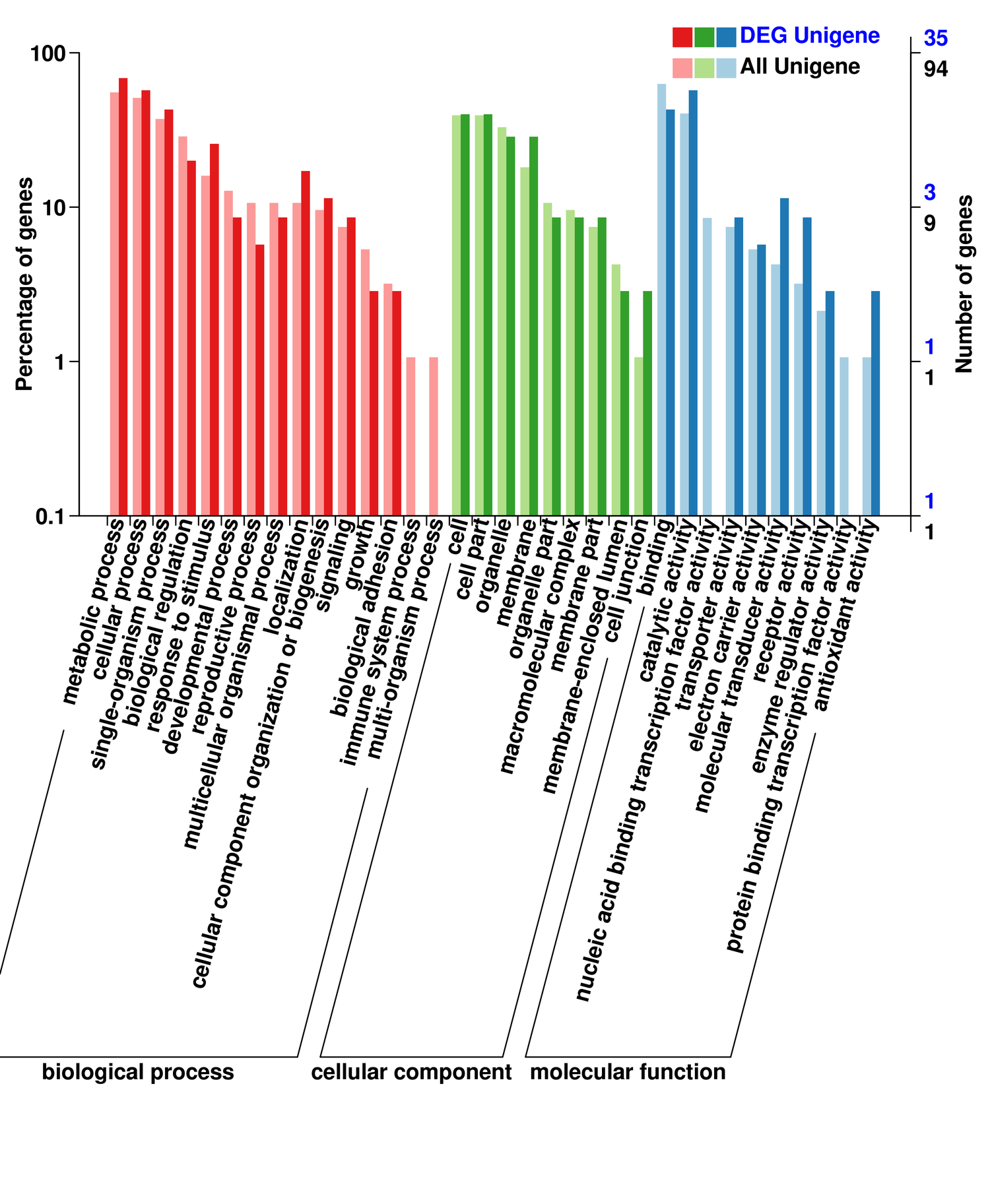

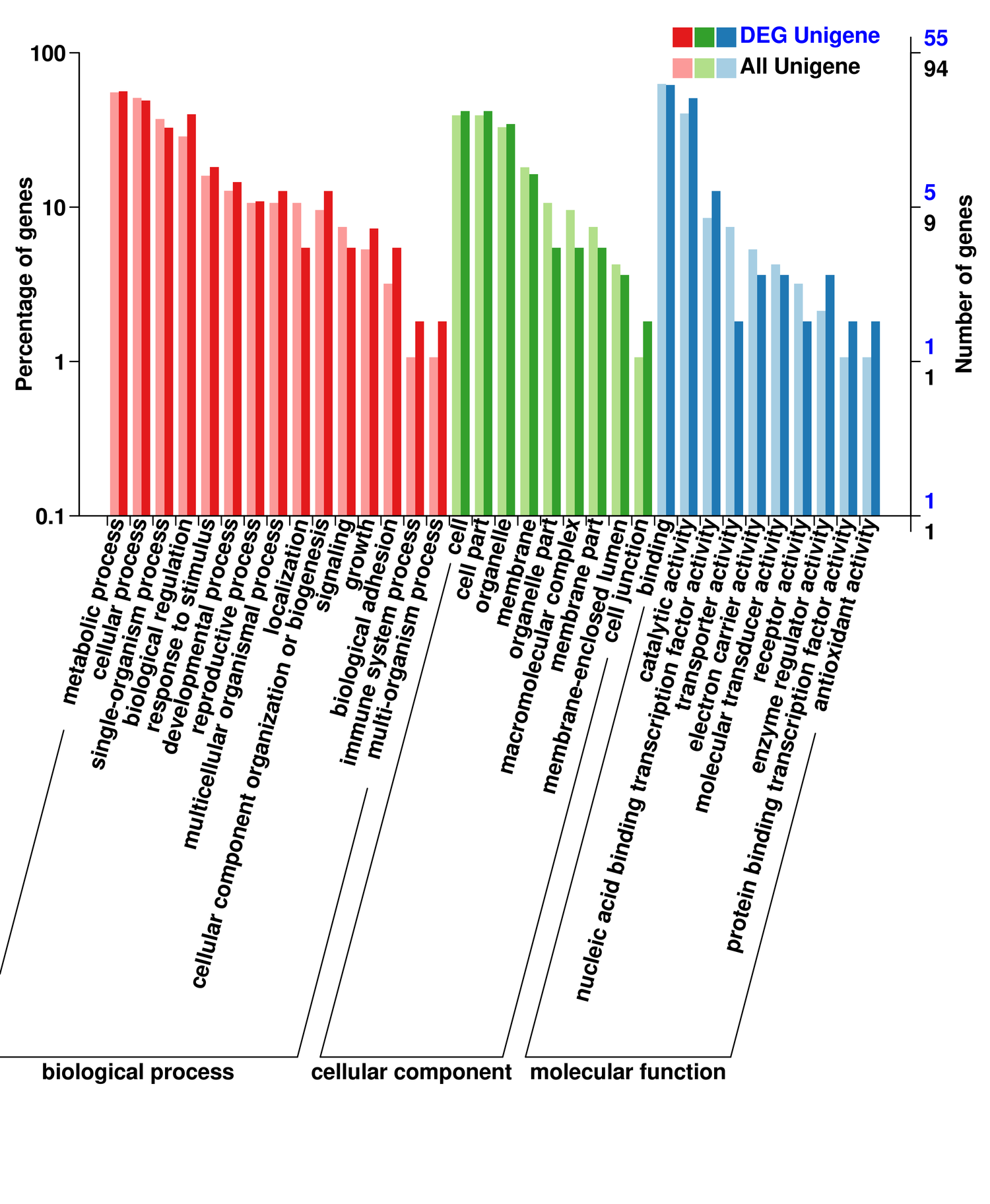


A

B

C

Figure S3

Supplement: Supplementary file 3 — Figure S3. GO enrichment of the targets of expressed miRNAs. (DOCX 4437 kb) [file 12864_2019_5770_MOESM3_ESM.docx]

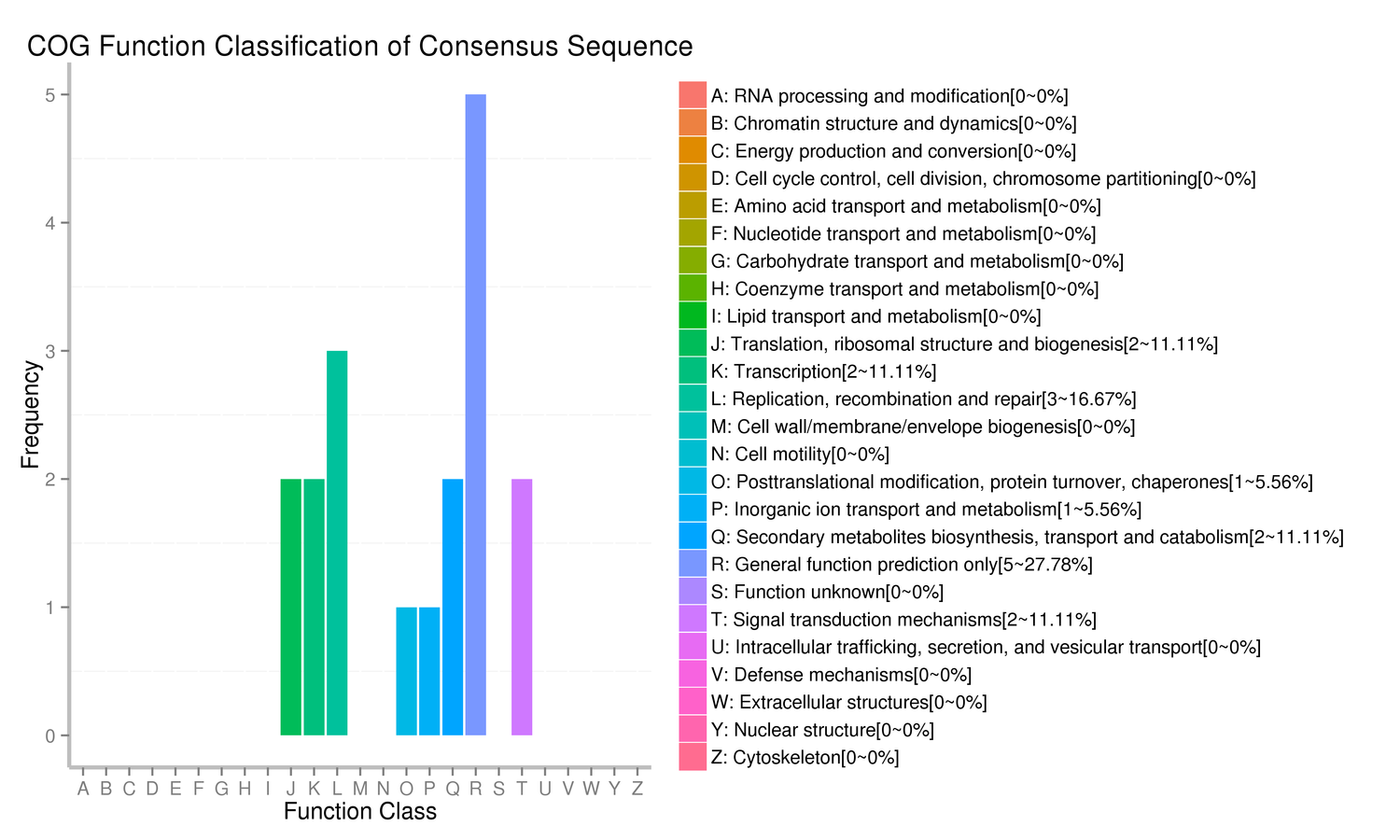


A


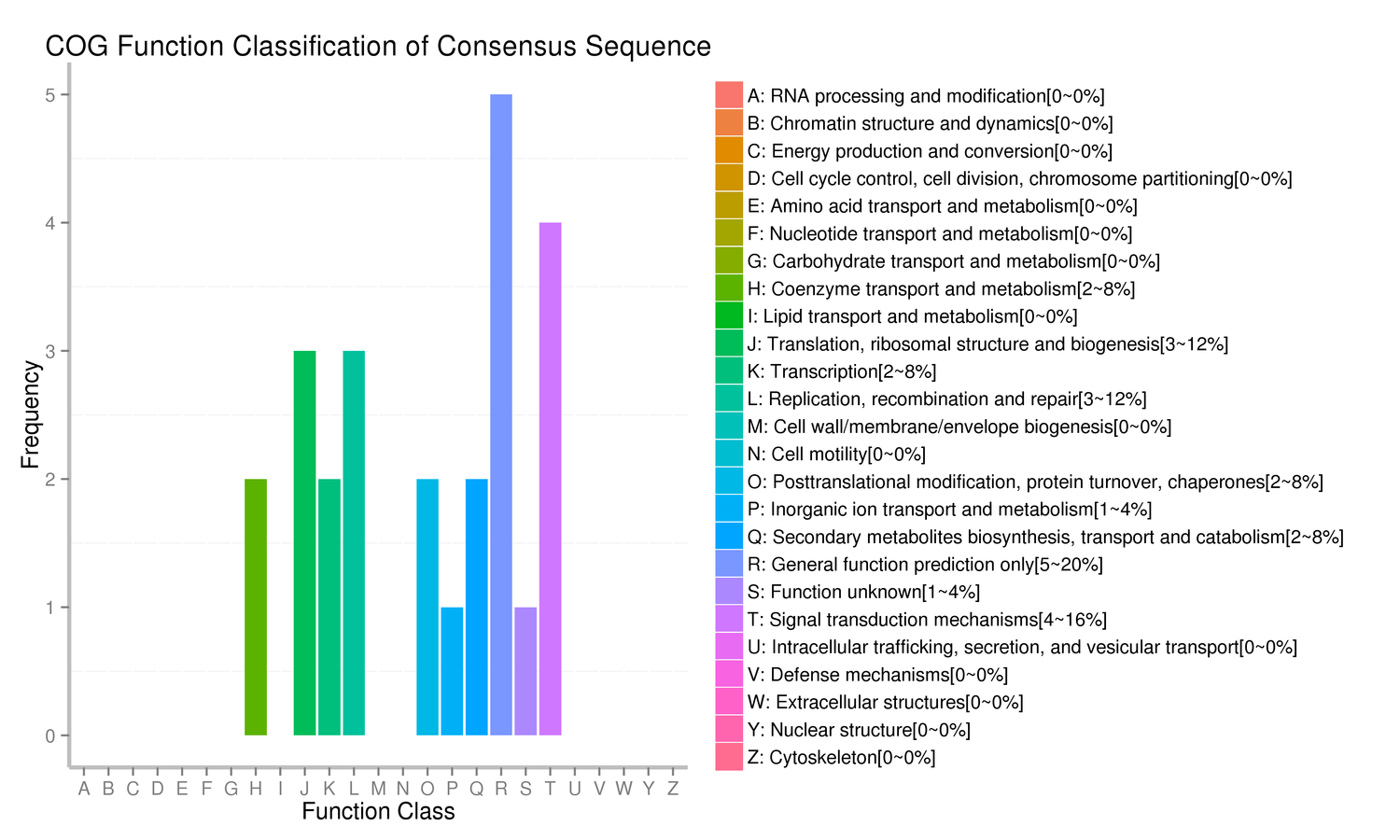


B


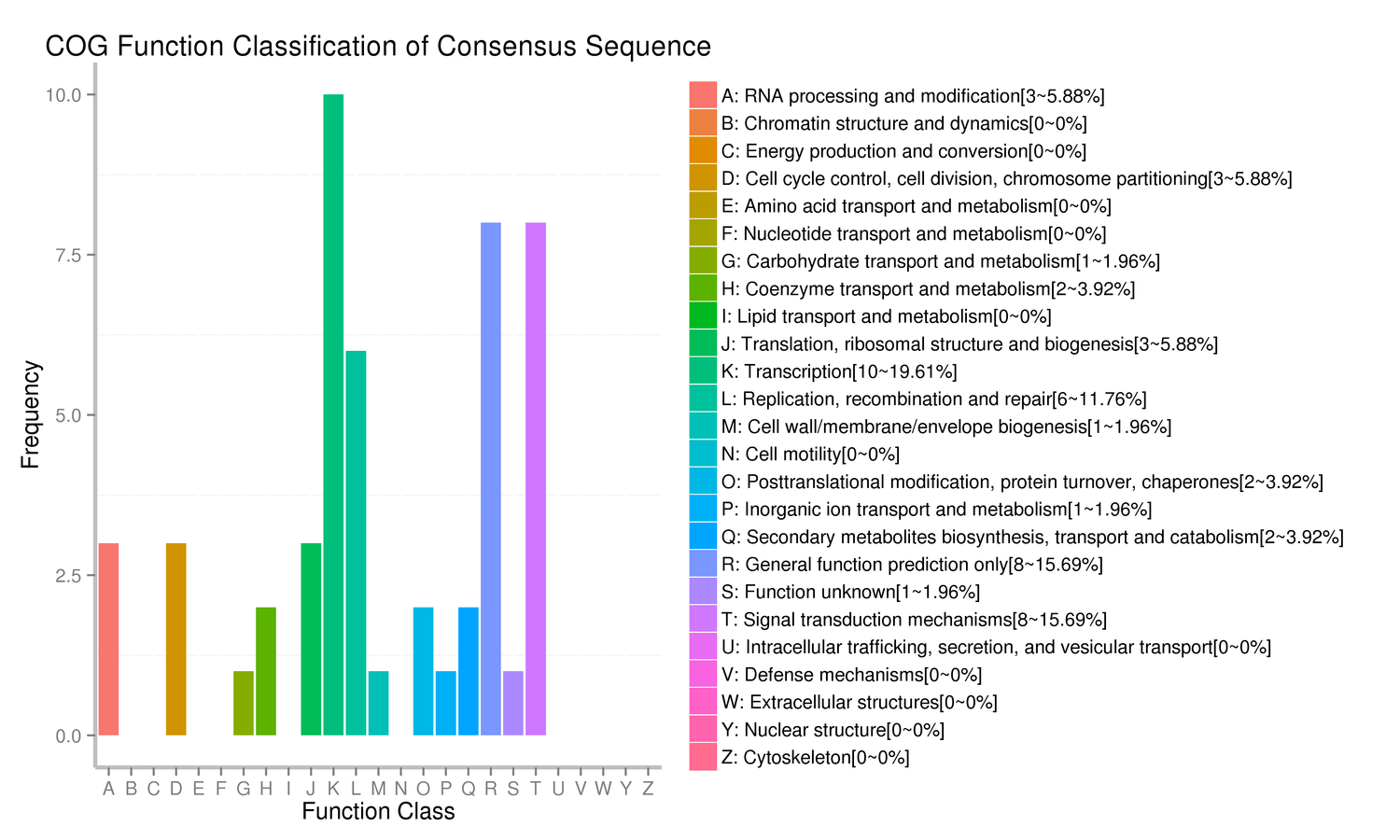


C

Figure S4

Supplement: Supplementary file 4 — Figure S4. COG functional classification of the target genes of differentially expressed miRNAs. (DOCX 2622 kb) [file 12864_2019_5770_MOESM4_ESM.docx]
